# The influence of genetic variants of sorafenib on clinical outcomes and toxic effects in patients with advanced renal cell carcinoma

**DOI:** 10.1038/srep20089

**Published:** 2016-02-02

**Authors:** Chao Qin, Qiang Cao, Pu Li, Shangqian Wang, Jian Wang, Meilin Wang, Haiyan Chu, Liqun Zhou, Xuesong Li, Dingwei Ye, Hailiang Zhang, Yiran Huang, Baijun Dong, Xiaofeng Sun, Qing Zou, Hongzhou Cai, Lijiang Sun, Jian Zhu, Fade Liu, Junbiao Ji, Li Cui, Xiaoxiang Wang, Hai Zhou, Hu Zhao, Bin Wu, Jianchun Chen, Minjun Jiang, Zhengdong Zhang, Pengfei Shao, Xiaobing Ju, Changjun Yin

**Affiliations:** 1 Department of Urology, the First Affiliated Hospital of Nanjing Medical University, Nanjing, China; 2 Department of Oncology, the First Affiliated Hospital of Nanjing Medical University, Nanjing, China; 3 Cancer Center of Nanjing Medical University, Department of Molecular & Genetic Toxicology, Nanjing Medical University, Nanjing, China; 4 Department of Urology, Peking University Fist Hospital, Beijing, China; 5 Department of Urology, Fu Dan University Shanghai Cancer Center, Shanghai, China; 6 Department of Urology, Shanghai Renji Hospital, Shanghai, China; 7 Department ofOncology, Jiangsu Cancer Hospital, Nanjing, China; 8 Department of Urology, Jiangsu Cancer Hospital, Nanjing, China; 9 Department of Urology, the Affiliated Hospital of Qing Dao University, Qiang Dao, China; 10 Department of Urology, Nantong Hospital of Traditional Chinese Medicine, Nantong, China; 11 Department of Urology, Nanjing Jiangning Hospital, Nanjing, China; 12 Department of Urology, the First People’s Hospital of Changzhou, Changzhou, China; 13 Department of Urology, Yangzhou NO.1 People’s Hospital, Yangzhou, China; 14 Department of Urology, Jiangyin People’s Hospital, Jiangyin, China; 15 Department of Urology, Wujiang NO.1 People’s Hospital, Wujiang, China

## Abstract

The purpose of the present study was to investigate whether genetic variants that influence angiogenesis and sorafenib pharmacokinetics are associated with clinical outcomes and toxic effects in advanced renal cell carcinoma patients treated with this drug. One hundred patients with advanced renal cell carcinoma were enrolled. Forty-two polymorphisms in 15 genes were selected for genotyping and analyzed for associations with progression-free survival, overall survival, and toxic effects. We found that rs1570360 in VEGF and rs2239702 in VEGFR2 were significantly associated with progression-free. Specifically, patients carrying the variant genotypes (AG + AA) of these two polymorphisms both had an unfavorable progression-free. In addition, compared with those with the rs2239702 GG genotype, patients with the AG + AA genotype suffered an unfavorable OS. We found that the VEGF rs2010963 CG + GG genotypes had a significantly increased risk of hand-foot syndrome, and the ABCB1 rs1045642 CT + TT genotypes had an increased risk of high blood pressure. Our results suggest that polymorphisms in VEGF and VEGFR2 are associated with sorafenib clinical outcomes, and polymorphisms in VEGF and ABCB1 are associated with sorafenib-related toxicities. Larger studies are warranted to validate our findings.

Renal cell cancer (RCC) is the predominant form of malignancy of the kidney (>80%) and accounts for about 3% of all adult malignancies, and 2% of all cancer deaths^1^. Although the detection rate for RCC has increased due to improved diagnostic techniques, it is estimated that approximately 25% of patients already have metastatic disease at the time of diagnosis, and 30% of surgically treated patients will develop metastases^2^,^3^. The outcome of patients with metastatic disease is dismal, as the 5-year survival rate is less than 10%, and RCC is highly resistant to chemotherapy and radiotherapy. Cytokine therapy, such as high-dose interleukin-2, interferon-alpha, or both combined, has been used to treat advanced RCC^4^, but the low response rate and substantial side effects make this treatment only appropriate for a small number of selected cases.

Because angiogenesis has a pivotal role in tumor growth, strategies in cancer drug development have focused on inhibiting this pathway. Aberrant angiogenesis is considered a hallmark of RCC, particularly for clear cell RCC (ccRCC). In the majority of ccRCC cases, the von Hippel–Lindau tumor suppressor gene VHL is functionally disrupted, leading to constitutive activation of hypoxia-inducible factors (HIFs) and subsequent induction of target genes such as vascular endothelial growth factor (VEGF)^5^.

Sorafenib is a multi-targeting tyrosine kinase inhibitor against VEGF receptors, platelet-derived growth factor (PDGF) receptors, FMS-like tyrosine kinase 3 (FLT-3), rearranged during transfection (RET) gene, KIT, and the RAF serine/threonine kinases^6^. Sorafenib was approved by the United States Food and Drug administration in December 2005 for treatment of metastatic RCC (mRCC), and is the first molecular-targeted drug used clinically for patients with advanced RCC in China. It seems to be more effective in patients of Chinese ethnicity than in western patients, and is well tolerated with a manageable toxicity profile^7^.

In clinical practice, there is a wide range of clinical outcomes and degrees of toxic effects experienced by patients treated with sorafenib^7^. Therefore, the identification of biomarkers that could predict clinical response and toxic effects would aid in maximizing therapeutic efficacy and avoid unnecessary costs and side effects. In exploring individual susceptibility, some genetic variations such as polymorphisms within candidate genes have shown promising potential as biomarkers of clinical response and toxicity associated with tyrosine kinase inhibitor treatment^8^,^9^,^10^. A number of studies have proposed that polymorphisms affecting the pharmacokinetic and pharmacodynamic pathways of sunitinib and pazopanib (two drugs also used for mRCC) alter the efficacy of these drugs in the treatment of mRCC patients^11^,^12^,^13^. However, there is a paucity of studies investigating individual susceptibility to sorafenib treatment.

In the present study, we analyzed 42 polymorphisms within 15 genes known to be involved in the sorafenib pharmacokinetic and pharmacodynamic pathways, and assessed the influence of these polymorphisms on clinical outcome and toxic effects in mRCC patients treated with sorafenib.

## Methods and Patients

The Institutional Review Board of Nanjing Medical University, Nanjing, China approved this prospective study. At recruitment, all participants involved in this study provided written informed consent. The methods were carried out in accordance with the approved guidelines.

Between October 2006 and April 2012, 107 patients with diagnosed mRCC were recruited from the following institutions in China: First Affiliated Hospital of Nanjing Medical University (41 patients) and Jiangsu Cancer Hospital (9 patients) in Nanjing; Fudan University Shanghai Cancer Center (9 patients) and RENJI Hospital (10 patients) in Shanghai; Peking University First Hospital (7 patients) in Beijing; First People’s Hospital of Changzhou (9 patients) in Changzhou; Affiliated Hospital of Medical College Qingdao University (10 patients) in Qingdao; and Yangzhou First People’s Hospital (3 patients) in Yangzhou; Nantong Hospital of Traditional Chinese Medicine in Nantong (2 patients); Nanjing Jiangning Hospital in Nanjing (2 patients); Jiangyin People’s Hospital (3 patients) in Jiangyin and Wujiang NO.1 People’s Hospital (2 patients). Patients were excluded from participating if they had central nervous system metastasis, age outside of 18–80 y, Karnofsky performance status < 80%, life expectancy < 3 months, rheumatoid disease, or acute inflammation.

All the included patients had newly diagnosed mRCC without previous chemotherapy or radiotherapy. Their disease was classified in accordance with the criteria of the World Health Organization and the 2002 American Joint Committee on Cancer tumor-node-metastasis (TNM) classification. The pathology slides from radical nephrectomy or core biopsy were independently reviewed by two urological pathologists, and were confirmed as ccRCC.

Sorafenib is the first molecular-targeted drug used for patients with mRCC in China. All the enrolled patients received sorafenib as their first-line treatment, which was given at 400 mg twice a day, orally, on a continual basis. Dosage modification to 800 mg twice a day was permitted if progression occurred and side effects could be tolerated. Treatment-related toxicity was graded using the National Cancer Institute Common Terminology Criteria for Adverse Events, version 3.0.

The patients were followed-up every 2 months at the outpatient department from the time of enrollment. Seven patients withdrew from the treatment for economic reasons and were excluded from further analysis. Computed tomography scans were obtained at baseline and after every 2 cycles (12 weeks) of therapy (on average) according to the treating physician’s discretion. Objective response was recorded per investigator assessment according to the Response Evaluation Criteria in Solid Tumors (RECIST) criteria. The primary endpoints for this analysis of prognostic factors to sorafenib therapy were progression-free survival (PFS) and overall survival (OS).

### Single nucleotide polymorphism (SNP) selection

We identified potentially functional polymorphisms in sorafenib pharmacokinetics genes (CYP3A4, CYP3A5, CYP1A1, CYP1A2, ABCB1, and ABCB2) or sorafenib pharmacodynamics genes (VEGF, VEGFR1, VEGFR2, VEGFR3, PDGFR, PDGFRB, IL8, HIF1A and EPAS1) according to the following criteria: (1) located in the 5′ flanking regions, 5′ untranslated region (UTR), 3′ UTR, or coding regions with amino acid changes; (2) minor allele frequency (MAF) > 5% in Chinese population. Besides, polymorphisms were reported to be significant in previous studies were also included in the present study. Finally, forty-two polymorphisms associated with sorafenib pharmacokinetics/pharmacodynamics were selected, as presented in online Table 1.

### DNA extraction and genotyping

Genomic DNA was extracted from peripheral blood using a QIAamp DNA Blood Maxi kit (Qiagen, Valencia, CA, USA). Depending on the characteristics of the SNP, either SNaPshot or PCR-sequencing were applied for genotyping (Table 1). The primers and probes used for genotyping are available upon request.

### Statistical analyses

The association between genotypes of SNPs and the rate of occurrence of side effects such as hand-food reaction, hypertension, and diarrhea were assessed by chi-squared test and logistic regression analysis. PFS was defined as the time from first administration of sorafenib to the first documentation of disease progression or death from any cause. OS was considered the time between the first day of application of sorafenib and the date of death or last date of follow-up. PFS and OS were estimated by the Kaplan-Meier method, and the log-rank test was used to compare different survival curves. Univariate or multivariate Cox regression analyses were performed to determine predictive factors of mRCC survival by estimating the crude hazard ratios (HRs), adjusted HRs, and their 95% confidence intervals (CIs), with adjustment for possible confounders. All analyses were performed with the software SAS 9.1.3 (SAS Institute, Cary, NC, USA) with two-sided *P*-values. *P* < 0.05 was considered significant.

## Results

### Patient characteristics

The median age of the patients was 56 years (Table 2). Seventy-three of the 100 patients were men. Metastatic organs included lungs (69%), kidney (20%), bone (29%), lymph node (29%), and brain and skin (6%). Thirty-three percent of the patients had only one metastatic site.

### Polymorphisms and OS of patients receiving sorafenib treatment

**Figure 1:**
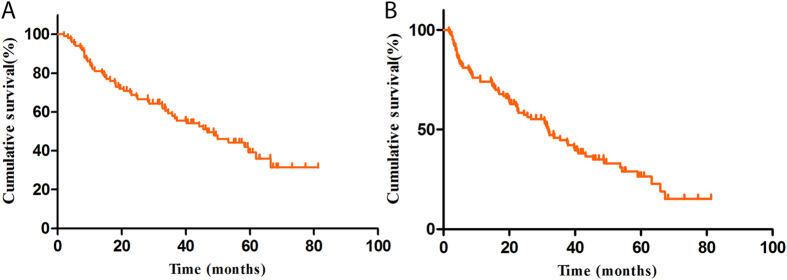
(**A**) OS of the patients treated with sorafenib; (**B**) PFS of patients treated with sorafenib.

**Figure 2:**
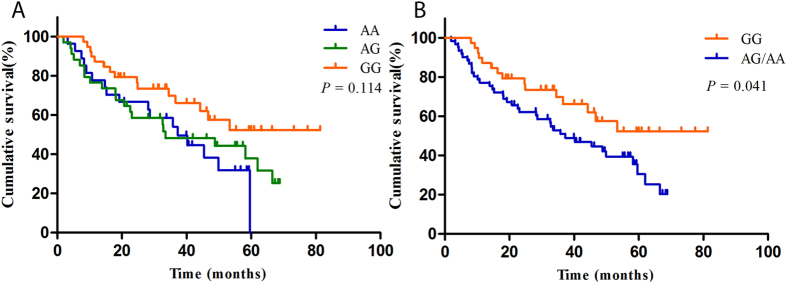
Kaplan-Meier curves show the OS and correlation with VEGFR2 rs2239702 polymorphism.

The median survival time (MST) of the cohort was 46.7 months (Fig. 1A). As shown in Table 3, we found that the SNP rs2239702, located in the 5′ UTR of VEGFR2, was significantly associated with unfavorable OS of the patients. Compared with the patients with the rs2239702 GG genotype, those with AG/AG had a shortened survival time (MST = 37.9 and 27.2, respectively, log-rank *P* = 0.041, Fig. 2).

### Polymorphisms and PFS of patients receiving Sorafenib treatment

**Figure 3:**
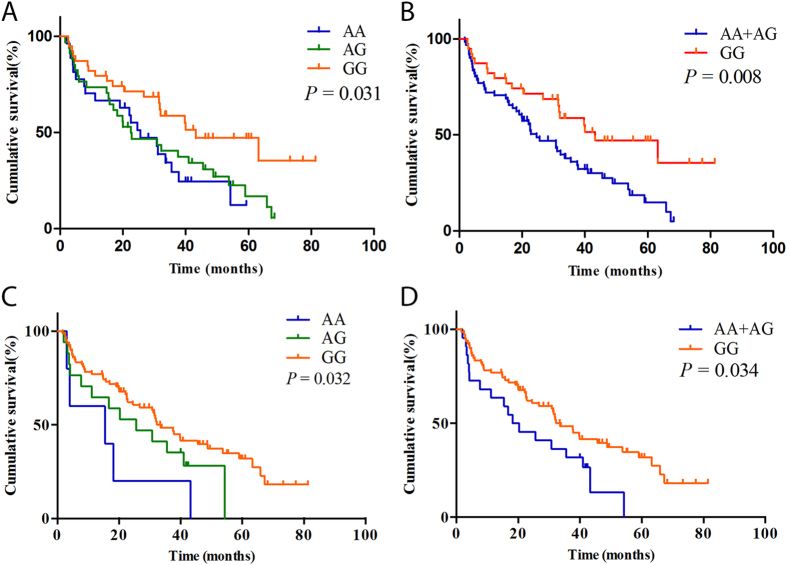
Kaplan-Meier curves show the PFS and correlation with VEGFR2 rs2239702 and VEGF rs1570360 polymorphisms.

The MST of the patients was 31.8 months (Fig. 1B). Two polymorphisms, VEGFR2 rs2239702 (as aforementioned) and another polymorphism located in the 5′ UTR of VEGF (rs1570360) were significantly associated with the PFS of patients (Table 4). The MST of the patients with the VEGFR2 GG genotype was 43.2 months, and for those with AG and AA genotypes were 22.5 and 25.2 months, respectively (log-rank *P* = 0.031). Furthermore, combined those with the variant genotypes (AG/GG), the patients with GG and AG/GG genotypes also had a significant difference in MST (MST = 43.2 and 24.5, respectively, log-rank *P* = 0.008, Fig. 3A,B). The VEGF rs1570360 was also associated with an inferior PFS. The MSTs for the patients with VEGF GG, AG, or AA, were 33.4, 20.3, and 4 months, respectively; and the difference was significant (log-rank *P* = 0.032). The difference in MST remained significant after combining the patients with variant genotypes AG/GG (MST = 33.4 and 18.2, for patients with GG and AG/AA genotypes, respectively; log-rank *P* = 0.034, Fig. 3C,D).

### Polymorphisms and toxic effects of sorafenib

Table 4 presents the frequency of toxic side effects experienced during treatment by patients stratified by the analyzed polymorphisms, including hand-food reaction, hypertension, and diarrhea. We found that there was a significant association between VEGF rs2010963 and the prevalence of hand-food reaction, which was more frequent in patients with the more unfavorable genotypes (rs2010963 CG and GG; *P* = 0.001, OR = 10.32, 95% CI = 2.10–68.63; online Table 5).

A polymorphism in ABCB1 (rs1045642) was found to be significantly associated with hypertension (Table 6), and patients with the more unfavorable genotypes (rs1045642 CT and CT/TT) experienced hand-food reaction more frequently (for TT cf. CC, *P* = 0.028, OR = 6.00, 95% CI = 1.22–29.53; for CT/TT cf. CC, *P* = 0.037, OR = 4.00, 95% CI = 1.09–14.67; Table 7)

## Discussion

Research into molecular-targeted treatment has been the focus for mRCC in recent years, especially with regard to outcomes and toxicity. Differences have been noted in RCC sufferers receiving sunitinib or pazopanib that are associated with genetic polymorphisms^11^,^12^,^13^. Sorafenib, the first drug chosen to cope with mRCC in China, is currently being used in a number of patients. Considering the crucial role of angiogenesis and pharmacokinetic-related genes in influencing the efficacy of targeted therapy for RCC, we investigated associations between key SNPs in these genes and the clinical outcomes of patients treated with sorafenib. We found that polymorphisms in VEGF and VEGFR2 were associated with clinical outcomes of sorafenib treatment, and polymorphisms in VEGF and ABCB1 were associated with sorafenib-related toxicities. To the best of our knowledge, this is the first study to explore the influence of genetic variants in angiogenesis and pharmacokinetic pathways on the clinical outcomes and toxic effects in mRCC patients treated with sorafenib. The study showed that genetic variations in VEGF and VEGFR2 were significantly associated with the PFS of RCC sufferers.

Carcinogenesis depends on the nutrition transported by blood vessels, the growth of which is determined by VEGF to a significant degree. For example, the inactivated VHL gene appears in nearly 60% of RCC patients. VHL protein, once inactivated, will decrease the number of degraded hypoxia-inducible factors [10878807]. Similarly, elevated levels of HIF-α can trigger the overexpression of VEGF, which then leads to stimulation of VEGFR and its downstream pathway, until tumor vessels generate^14^. Therefore, genetic variations in genes responsible for angiogenesis are crucial to the development of ccRCC, patients’ prognosis, and the effect of drugs in targeted therapy. The crucial role of angiogenic signaling pathway in cancer has led to the development of medicines that inhibit VEGF receptor-targeted tyrosine kinase and have proven benefits in clinic use. The efficacy of these tyrosine kinase inhibitors in inhibiting several receptor tyrosine kinases in the angiogenic signaling pathway may be responsible for their efficacy.

In our previous studies, we found that VHL and HIF1A polymorphisms may contribute jointly to influence the progression and prognosis of RCC^15^. Garcia-Donas *et al*.^16^ found that VEGFR rs307826 and rs307821 as well as CYP3A5 rs776746 were significantly correlated with the PFS of RCC sufferers treated with sunitinib in a European population. As suggested by Kim *et al*.^17^, in metastatic ccRCC patients treated with sunitinib a combination of VEGF SNP 936 and VEGFR2 SNP 889 genotypes is associated with OS. In our study, we found that rs2239702, located in the 5′UTR of VEGFR2, was significantly associated with unfavorable OS of the patients. The median OS of rs2239702 GG carriers was significantly higher than that of GA or AA carriers.

In the analysis of PFS, two polymorphisms, VEGFR2 rs2239702 and another polymorphism located in the 5′UTR of VEGF (rs1570360), were significantly associated with PFS of the patients. Regarding VEGF rs1570360, the PFS of patients carrying the A allele was significantly shorter than the PFS of those carrying the G allele. The PFS of AA carriers was 33.4 months, AG 20.33 months, and AA 4 months. For VEGFR2 rs2239702, it was found that the PFS of patients carrying the A allele was significantly shorter than for those carrying the G allele. These data suggest that alternative treatment approaches for patients with these genetic variants should be promoted.

Although the nature and incidence of adverse events related to sorafenib are currently well recognized and described, data regarding determinants of toxicity are still scarce. In our study, we found that there was a significant association between VEGF rs2010963 and the prevalence of hand-foot reaction. Although there has been little study addressing the role of the VEGF pathway in the pathophysiology of hand-foot reaction, Azad *et al*.^18^ provided evidence that inhibition of the VEGF pathway may be an important factor in sorafenib-related hand-foot reaction. Since rs2010963 is located in the 5′-UTR region of VEGF, it may alter VEGF expression by modulating promoter activity. Assuming that VEGF rs2010963 leads to decreased gene expression, it may mimic inhibition of VEGF, and then confer susceptibility to hand-foot reaction. However, this deduction should be rigorously investigated in a future study.

In the present study, another polymorphism located in the exon region of ABCB1 (rs1045642) was found to be significantly associated with hypertension. ABCB1 is located in human chromosome 7 q21.1. ABCB1, a dominate gene in the human multidrug resistance gene family, can be regulated for its expression is decided by different factors^12^. Hoffmeyer *et al*.^19^ reported that the protein level of *ABCB1* and the plasma drug levels of P-glycoprotein substrates were affected by polymorphisms in ABCB1. Their study revealed that P-glycoprotein levels in the duodenum of patients carrying 3435 CC were at least two-fold higher than that of patients carrying 3435 TT. The plasma drug levels dropped significantly when digoxin was given orally. These researches prove that high levels of P-glycoprotein affects drug absorption in the small intestine.

In 2007, Ebid *et al*.^20^ tested the plasma phenytoin level of epilepsy sufferers with ABCB1 C3435T. Polymorphisms in ABCB1 may result in individual differences in bioavailability and toxicity when drugs are taken orally, by changing drug absorption in the small intestine. Similar results have been found in research on sunitinib. Hand-foot reaction is associated with the copy sequence of haplotype TTT in ABCB1 (3435c/T, 1235 C/t, 2677 G/T), indicating that genetic variation in ABCB1 can affect toxicity and its severity when molecular-targeted drugs are administered^11^. Our present study showed that genetic variants that affect angiogenesis and pharmacokinetic pathways could also influence the occurrence of sorafenib-related toxicity in mRCC patients.

Our results herein suggest that polymorphisms in VEGF and VEGFR2 are associated with clinical outcomes of sorafenib treatment, and polymorphisms in VEGF and ABCB1 are associated with sorafenib-related toxicities. As limitation of small sample size and lack of multiple comparison exit in the present study, the initial findings should be verified in the future studies. If confirmed, these genetic variants could provide the basis for individualized mRCC treatment.

## Additional Information

**How to cite this article**: Qin, C. *et al*. The influence of genetic variants of sorafenib on clinical outcomes and toxic effects in patients with advanced renal cell carcinoma. *Sci. Rep.* **6**, 20089; doi: 10.1038/srep20089 (2016).

## Figures and Tables

**Table 1:** Genotyping methods for selected polymorphisms (should be online only).

**Gene**		**Mutation**	MAF*	**Genotyping methods**
VEGF	rs699947	C > A	0.278	SNaPshot
	rs2010963	G > C	0.352	PCR-sequencing
	rs3025040	C > T	0.178	SNaPshot
	rs10434	G > A	0.189	SNaPshot
	rs1570360	A > G	0.233	PCR-sequencing
VEGFR1	rs9554314	A > C	0.322	SNaPshot
	rs664393	G > A	0.244	SNaPshot
VEGFR2	rs1870377	A > T	0.467	SNaPshot
	rs2305948	C > T	0.156	SNaPshot
	rs7667298	C > T	0.284	SNaPshot
	rs2239702	G > A	0.111	SNaPshot
VEGFR3	rs448012	C > G	0.483	SNaPshot
	rs72816988	G > A	0.079	SNaPshot
	rs1382302	A > G	0.430	SNaPshot
	rs59769082	T > G	0.492	SNaPshot
	rs13187322	A > G	0.475	PCR-RFLP
PDGFRA	rs35597368	T > C	0.183	PCR-sequencing
	rs3690	A > C	0.188	SNaPshot
PDGFRB	rs2229562	T > C	0.071	SNaPshot
	rs2302273	G > A	0.081	PCR-sequencing
	rs3828610	A > C	0.453	SNaPshot
IL8	rs1126647	A > T	0.308	PCR-sequencing
	rs4073	A > T	0.333	SNaPshot
CYP3A4	rs2740574	C > T	1	SNaPshot
	rs12721626	G > A	<0.05	SNaPshot
CYP3A5	rs776746	C > T	0.337	SNaPshot
	rs17161788	T > C	0.017	SNaPshot
	rs15524	T > C	0.303	SNaPshot
CYP1A1	rs1048943	A > G	0.23	PCR-sequencing
	rs4646422	G > A	0.149	SNaPshot
CYP1A2	rs2069522	T > C	0.067	SNaPshot
ABCB1	rs1045642	A > G	0.417	PCR-sequencing
	rs1128503	A > G	0.408	PCR-sequencing
	rs2032582	A > C	0.439	SNaPshot
ABCB2	rs2231142	C > A	0.289	PCR-sequencing
	rs2231137	C > T	0.289	PCR-sequencing
	rs2622604	T > C	0.207	SNaPshot
HIF1A	rs11549465	C > T	0.044	PCR-sequencing
	rs11549467	G > A	0.070	SNaPshot
	rs2057482	T > C	0.244	SNaPshot
EPAS1	rs59901247	A > C	0.175	PCR-sequencing
	rs17034935	C > T	0.085	PCR-sequencing

^*^MAF, minor allele frequency; PCR, Polymerase chain reaction.

**Table 2:** Clinical characteristics of the patients (should be online only).

		**Patients (n = 100)**
Age (years)		56 ± 12
Gender	Male	73 (73%)
	Female	27 (27%)
ECOG* score	0	24 (24%)
	1	57 (57%)
	2	16 (16%)
	3	3 (3%)
Hand surgery treatment		81 (81%)
Metastatic location	Lung	69 (69%)
	Kidney	20 (20%)
	Bone	29 (29%)
	Lymph node	48 (48%)
	Brain and Skin	6 (6%)
Number of metastatic sites	1	33 (33%)
	2	32 (32%)
	3	24 (24%)
	4	4 (4%)
	5	6 (6%)
	6	1 (1%)

^*^Eastern Cooperative Oncology Group.

**Table 3:** Association between VEGFR2 rs2239702 polymorphisms and OS of the patients.

**Rs2239702**	**Deaths**	**All patients**	MST*	**Log-rank** ***P***
GG	15	39	37.9	0.114
AG	21	34	33.5	
AA	17	27	37.2	
GG	15	39	37.9	0.041
AG + AG	38	61	27.2	

^*^Mean survival time was calculated since the deaths were less than 50%.

**Table 4:** Association between VEGFR2 rs2239702 and VEGF rs1570360 polymorphisms and the PFS of the patients.

**VEGFR2**	**Progression**	**All patients**	MST*	**Log-rank** ***P***
Rs2239702				
GG	19	39	43.2	0.031
AG	28	34	22.5	
AA	20	27	25.2	
GG	19	39	43.2	0.008
AG + AA	48	61	24.5	
VEGF Rs1570360				
GG	49	78	33.4	0.032
AG	13	17	20.33	
AA	5	5	4	
GG	49	78	33.4	0.034
AG + AA	18	22	18.2	

^*^Mean survival time.

**Table 5:** Associations between genetic polymorphisms and toxic effects of sorafenib.

	**Hand-foot reaction**		**Hypertension**		**Diarrhea**	
	**OR**	***P***	**OR**	***P***	**OR**	***P***
VEGF rs699947	1.67 (0.42–6.71)	0.468	0.88 (0.32–2.43)	0.807	2.17 (0.94–5.01)	0.068
VEGF rs2010963	10.32 (2.67–40.03)	0.001	0.58 (0.22–1.55)	0.278	1.06 (0.46–2.46)	0.883
VEGF rs3025040	2.70 (0.55–13.20)	0.220	0.97 (0.35–2.69)	0.958	1.72 (0.74–3.97)	0.206
VEGF rs1570360	1.26 (0.25–6.28)	0.778	1.28 (0.41–3.98)	0.673	1.52 (0.58–3.96)	0.394
VEGFR1 rs9554314	1.98 (0.54–7.22)	0.299	0.79 (0.15–4.11)	0.777	1.09 (0.48–2.47)	0.828
VEGFR1 rs664393	0.92 (0.25–3.36)	0.896	0.41 (0.13–1.33)	0.137	2.32 (0.87–6.19)	0.094
VEGFR1 rs448012	2.19 (0.62–7.70)	0.223	0.87 (0.32–2.36)	0.789	0.95 (0.41–2.19)	0.901
VEGFR3 rs72816988	0.28 (0.06–1.23)	0.091	0.86 (0.17–4.25)	0.849	0.39 (0.08–1.91)	0.247
VEGFR3 rs1382302	1.09 (0.30–4.00)	0.896	0.58 (0.22–1.56)	0.280	0.95 (0.41–2.24)	0.916
VEGFR3 rs13187322	2.07 (0.57–7.53)	0.270	1.47 (0.55–3.94)	0.448	1.04 (0.46–2.34)	0.932
VEGFR2 rs1870377	2.00 (0.57–7.03)	0.280	0.58 (0.22–1.55)	0.278	0.89 (0.39–2.04)	0.781
VEGFR2 rs2305948	1.95 (0.40–9.58)	0.412	0.78 (0.26–2.38)	0.665	1.27 (0.52–3.07)	0.599
VEGFR2 rs7667298	1.39 (0.38–5.12)	0.623	0.99 (0.34–2.87)	0.992	0.84 (0.35–2.03)	0.706
VEGFR2 rs2239702	1.61 (0.46–5.64)	0.457	0.71 (0.26–1.94)	0.506	0.59 (0.26–1.33)	0.202
PDGFRArs35597368	0.62 (0.15–2.55)	0.504	1.48 (0.47–4.66)	0.504	1.09 (0.40–3.02)	0.864
PDGFRA rs3690	0.32 (0.08–1.25)	0.101	2.24 (0.19–25.96)	0.520	1.00 (0.34–2.87)	0.984
PDGFRB rs2229562	1.11 (0.22–5.57)	0.899	0.68 (0.21–2.13)	0.504	0.83 (0.29–2.36)	0.725
PDGFRB rs2302273	1.58 (0.32–7.84)	0.573	1.35 (0.46–3.95)	0.587	0.69 (0.26–1.83)	0.452
PDGFRB rs3828610	1.22 (0.35–4.27)	0.758	0.82 (0.31–2.19)	0.696	1.00 (0.44–2.28)	0.994
IL8 rs1126647	2.92 (0.80–10.66)	0.106	1.01 (0.37–2.72)	0.985	1.63 (0.69–3.83)	0.261
IL8 rs4073	1.33 (0.38–4.67)	0.657	1.25 (0.45–3.45)	0.666	1.54 (0.66–3.63)	0.320
CYP3A5 rs776746	1.02 (0.29–3.55)	0.981	0.50 (0.12–2.11)	0.343	0.67 (0.18–2.56)	0.559
CYP3A5 rs15524	1.02 (0.29–3.55)	0.981	0.50 (0.12–2.11)	0.343	0.67 (0.18–2.56)	0.559
CYP1A1 rs1048943	1.67 (0.42–6.71)	0.468	2.44 (0.91–6.56)	0.076	0.88 (0.38–2.04)	0.761
CYP1A1 rs4646422	0.38 (0.11–1.34)	0.132	0.43 (0.13–1.40)	0.160	1.35 (0.58–3.15)	0.493
CYP1A2 rs2069522	1.71 (0.20–14.40)	0.623	1.72 (0.49–6.12)	0.397	0.29 (0.06–1.36)	0.116
ABCB1 rs1045642	0.35 (0.07–1.73)	0.200	4.00 (1.09–14.67)	0.037	1.08 (0.46–2.51)	0.866
ABCB1 rs1128503	0.70 (0.19–2.57)	0.595	1.22 (0.45–3.28)	0.695	1.18 (0.52–2.69)	0.696
ABCB2 rs2231142	0.82 (0.23–2.88)	0.758	0.94 (0.35–2.54)	0.910	1.43 (0.63–3.25)	0.395
ABCB2 rs2231137	0.44 (0.09–2.18)	0.317	1.11 (0.38–3.19)	0.850	0.45 (0.19–1.05)	0.064
ABCB2 rs2622604	0.44 (0.12–1.62)	0.219	0.92 (0.10–8.66)	0.939	0.53 (0.23–1.23)	0.139
HIF1Ars2057482	5.48 (0.67–44.68)	0.112	0.86 (0.30–2.46)	0.775	0.80 (0.33–1.93)	0.620
EPAS1 rs59901247	0.49 (0.13–1.83)	0.290	0.64 (0.36–3.45)	0.848	0.79 (0.30–2.13)	0.644
EPAS1 rs17034935	1.50 (0.16–13.75)	0.720	1.56 (0.16–12.39)	0.758	0.60 (0.13–2.84)	0.519

**Table 6:** Association between ABCB1 rs1045642 and hypertension of patients treated with sorafenib.

	**Normal, n (%)**	**Severe, n (%)**	**Adjusted** P*	OR (95% CI)*
CC	36 (40.4)	3 (15.0)	—	1.00 (reference)
CT	41 (47.1)	12 (60.0)	0.067	3.51 (0.92–13.44)
TT	10 (11.5)	5 (25.0)	0.028	6.00 (1.22–29.53)
CT + TT	51 (58.6)	17 (85.0)	0.037	4.00 (1.09–14.67)

^*^Derived from logistic regression model.

**Table 7:** Association between VEGFA rs2010963 and hand-foot reaction of patients treated with sorafenib.

	**Normal, n (%)**	**Severe, n (%)**	**Adjusted** P*	OR (95% CI)*
CC	6 (54.6)	10 (10.4)	—	1.00 (reference)
CG	3 (27.3)	46 (47.9)	0.005	9.20 (1.96–43.15)
GG	2 (18.2)	40 (41.7)	0.005	12.00 (2.10–68.63)
CG + GG	5 (45.5)	86 (89.6)	0.001	10.32 (2.67–40.03)

^*^Derived from logistic regression model. Adjusted for age and gender.
